# Optimal fiber mix and prediction model for compressive strength of hybrid fiber reinforced concrete

**DOI:** 10.1371/journal.pone.0318713

**Published:** 2025-02-19

**Authors:** Ping Gao, Weihong Xie, Yongqiang Ling, Hongfa Xu

**Affiliations:** College of Building Engineering, Shanghai Zhongqiao Vocational and Technical University, Shanghai, China; University of Science and Technology Beijing, CHINA

## Abstract

Incorporating single or mixed types of fibers into concrete is an effective method for enhancing its performance. This study aimed to investigate the optimal fiber mix for hybrid fiber reinforced concrete (HFRC) that includes end-hooked steel fibers (ESF), corrugated steel fibers (CSF), and polypropylene fibers (PF), and to predict its compressive strength. To this end, three combinations of HFRC were designed: ESF and CSF (EC-HFRC), ESF and PF (EP-HFRC), and CSF and PF (CP-HFRC), respectively. Plain concrete and single-fiber reinforced concrete with the same mix proportions were also designed for comparison. The compressive strength test results demonstrate slight to moderate enhancement in the concrete’s compressive strength when hybrid fibers are employed. The hybrid effects observed in both EP-HFRC and CP-HFRC are superior to those of EC-HFRC. The optimal fiber mixing combinations are identified as 0.2% PF combined with 1.5% ESF for EP-HFRC and 0.2% PF along with 1.5% CSF for CP-HFRC. Compared with single steel fiber admixture and no fiber admixture, the mixing of hybrid fibers can significantly impact the failure mode of concrete. A novel mathematical model, based on the theory of composite mechanics, has been proposed to accurately predict the compressive strength of single steel fiber reinforced concrete and EC-HFRC, as evidenced by its close alignment with experimental data. The results of this paper provide substantial theoretical support for the design and optimization of HFRC.

## 1 Introduction

Concrete, as a widely used building material, has always been a focus of research and industry on optimizing and enhancing its performance. Recent studies have demonstrated that the integration of fibers into concrete leads to substantial improvements in numerous concrete properties, including but not limited to: mechanical strength [1–3], crack resistance [4–5], and impact resistance [6–7]. According to specific requirements, various types of fibers—encompassing four primary classifications (steel, glass, synthetic and natural)—can be combined in hybrid fiber reinforced concrete (HFRC) to achieve the optimal reinforcement.

The incorporation of steel fibers has been proved to significantly enhance the tensile strength, fracture energy and post-cracking load-bearing capacity of the resulting composite material [8–9]. Wu et al. [10–11] investigated the steel fiber concrete with different ratios and shape of fiber and found that the compressive and flexural strength of concrete were enhanced with the increase of steel fiber mixing ratio, and the deformed steel fibers significantly improved flexural strength, toughness and fiber-matrix bond strength of concrete relative to straight steel fibers.

Although steel fibers with high modulus and high strength can significantly boost concrete’s strength, their innate brittleness prevents concrete from becoming more ductile [12]. Low strength fibers like polypropylene are better at improving ductility and minimizing cracking [13]. The effectiveness of polypropylene fibers (PF) in enhancing post-cracking ductility depends on its better bond performance with the concrete matrix [14]. The use of synthetic fiber in concrete can help reduce the width of cracks while simultaneously increasing split tensile strength [15]. Hassan et al. [16] investigated that an increasing trend of splitting tensile strength was followed with increasing hybrid PF volume rate. The mix with the highest fiber volume rate of 0.9% macro and 0.1% micro PF yielded the highest increase by 29% compared to the control mix. The research conducted by Wang et al. [17] demonstrated that the hybridization of steel fibers and PF could significantly influence the strength degradation of recycled aggregate concrete after the peak compressive strength, and effectively ameliorate the brittleness of the concrete.

Despite of fiber type, fiber mixing rate and size have different degrees of influence on the mechanical properties of concrete [18–19]. Zhang et al. [20] investigated HFRC with different fiber mixing ratios and fiber size, and found that the use of 1.0% hybrid fibers (steel fiber, polyvinyl alcohol fiber, calcium carbonate whisker and basalt fiber included) resulted in the best mechanical properties, and the compressive and flexural strength of HFRC increased most significantly when the fiber lengths were 10 mm and 15 mm.

While significant progress has been achieved in the research of fiber-reinforced concrete (FRC), it is evident that the current study of HFRC is primarily focused on its fundamental mechanical properties, with a limited investigation into hybrid fiber effects and the optimal fiber mix. Consequently, it is imperative to embark on a research endeavor focused on the optimized design of HFRC fibers, taking into account the various types of hybrid fibers, their reinforcement coefficients, and effect coefficients. This constitutes a pivotal aspect of the present study.

In terms of compressive strength prediction models for fiber-reinforced concrete and hybrid fiber-reinforced concrete, there are relatively few researchers delving into this area. Abadel et al. [21] summarized the research findings of several scholars, indicating a linear relationship between compressive strength and fiber reinforcement index. Feng et al. [22] based on regression analysis of experimental data, proposed a parabolic strength prediction model. Ganesh et al. [23] conducted an analysis using artificial neural networks to predict the compressive strength of fiber-reinforced geopolymer concrete based on three different input conditions. Wang et al. [24] employed CT scanning technology to observe the microscopic crack development process of steel fiber-reinforced recycled aggregate concrete, expounded the damage development mechanism of the concrete, and proposed prediction equations for the stress and strain relationship of the reinforced concrete at different loading stages. Clearly, research on compressive strength prediction models for FRC remains insufficient. Although intelligent prediction is a current hotspot, there are still deficiencies in facilitating rapid design. Therefore, proposing a novel strength prediction model is of great significance. This also constitutes one of the research contents of this paper.

In order to further explore the synergistic effect of steel and synthetic fibers for enhancing the mechanical properties of concrete, two high modulus of elasticity steel fibers and one low modulus of elasticity polypropylene fiber were selected for combination in this paper. Through the carefully designed test scheme, the compressive strength and damage degree of single-fiber reinforced concrete (SFRC), plain concrete (PC) and the three fiber mixed HFRC specimens under the same conditions were compared aiming to reveal the optimization mechanism of the mixed fiber system on the mechanical properties of concrete. Furthermore, based on the theory of composite material mechanics, a predictive model of the ultimate compressive strength of FRC have been derived, which aligns well with the experimental results.

The incorporation of fibers not only diminish the quantity of conventional steel bars within the segments while preserving mechanical attributes of the concrete, but also fully exploits the mechanical properties of the fibers to achieve economic advantages. The research in this paper enriches and complements the previous studies, and is an important reference for the selection of fiber types and the determination of mixing ratios in engineering applications.

## 2 Methods and materials

### 2.1 Materials and mix proportions

**Fig 1:**
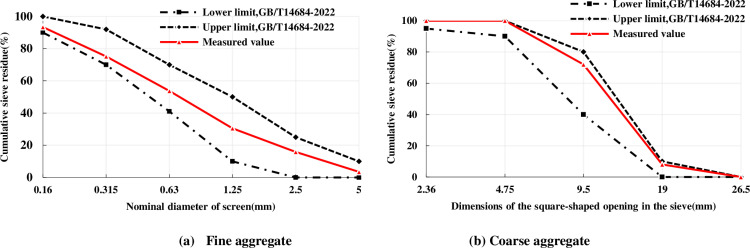
Grading curves of coarse and fine aggregates. (a) Grading curve of fine aggregate. (b) Grading curve of coarse aggregate.

This study was conducted in five groups, using identical basic mix proportions as the concrete matrix. According to the Chinese standards, the composite Portland cement 42.5 [25] was used as the cementing material from Xuzhou. The fine aggregate chosen is river sand, with a fineness modulus of 2.6, classified as medium sand, and a nominal particle size not exceeding 5.0 mm. The inspection of various indicators met the requirements of Class II sand standard for construction [26]. The natural crushed stone used for coarse aggregate, with a particle size of 5-20mm, meets the technical requirements of Class I [27] in terms of its physical indices such as needle and flake content, crushing value, particle size distribution, and harmful impurities. A sieve analysis was conducted on both the fine and coarse aggregates, with the resulting gradation curves presented in Fig 1. It was found that the gradation curves comply with the requirements of the Chinese standards GB/T14684-2022 [26] and GB/T14685-2011 [27], respectively.

**Fig 2:**
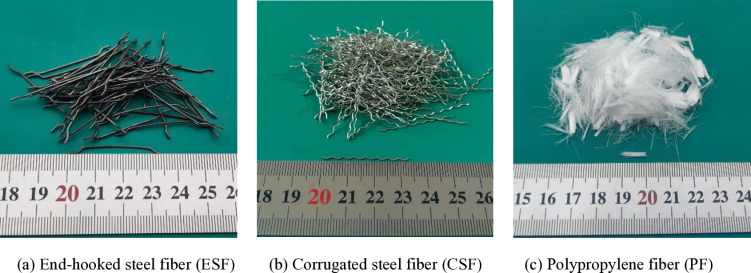
Photographs of fiber. (a) depicts ESF with a length of 35mm. (b) shows CSF with a length of 34mm. (c) shows PF with a length of 9 mm.

This paper examines the impact of three distinct types of fibers on concrete properties: end-hooked steel fibers (ESF), corrugated steel fibers (CSF) and PF. ESF and CSF are produced by Shandong Lubang Steel Fiber Co., Ltd. PF is bundled monofilament polypropylene fiber, produced by Shanghai Mengmeijia Chemical Technology Co., Ltd. Fig 2 and Table 1 present the characteristics of these three fiber types.

**Table 1:** Geometry and properties of ESF, CSF and PF.

Fiber class	Fiber type	Length (mm)	Diameter (mm)	Aspect ratio	Density (g/cm3)	Tensile strength (MPa)	Elastic modulus (GPa)
Macro	**ESF**	35	0.75	46.67	7.86	≥ 600	200
	**CSF**	34	0.5	68	7.86	≥ 600	200
Micro	**PF**	9	0.05	180	0.91	≥ 250	3.79

In accordance with the specifications set forth in the Chinese code JGJ55-2011 [28], a concrete matrix with a compressive strength of no less than 60 MPa has been formulated. The high-performance water reducer was used in this test in order to improve the workability of concrete. Based on the Chinese code JGJ55-2011 [28], the concrete matrix mix proportions were presented in Table 2. The matrix was reinforced at variable fiber addition rates according to JGJ/T 221-2010 [29]. The fiber incorporation of the five Groups (O, P, Q, R and S) were designed as presented in Table 3.

**Table 2:** Concrete matrix mix proportions (kg/m^3^).

Cement	Fine aggregate	Coarse aggregate	Water	Water reducer
486	645	1099	170	10

**Table 3:** Design of fiber incorporation in five groups.

Groups	Group no.	Fiber content (%)		
		V_ESF_^a^	V_CSF_^a^	V_PF_^a^
**O**	**O**	–	–	–
**P**	**P-1**	0.5	0.5	–
	**P-2**	0.5	1	–
	**P-3**	0.5	1.5	–
	**P-4**	1	0.5	–
	**P-5**	1	1	–
	**P-6**	1	1.5	–
	**P-7**	1.5	0.5	–
	**P-8**	1.5	1	–
	**P-9**	1.5	1.5	–
**Q**	**Q-1**	0.5	–	0.1
	**Q-2**	0.5	–	0.2
	**Q-3**	0.5	–	0.3
	**Q-4**	1	–	0.1
	**Q-5**	1	–	0.2
	**Q-6**	1	–	0.3
	**Q-7**	1.5	–	0.1
	**Q-8**	1.5	–	0.2
	**Q-9**	1.5	–	0.3
**R**	**R-1**	–	0.5	0.1
	**R-2**	–	0.5	0.2
	**R-3**	–	0.5	0.3
	**R-4**	–	1	0.1
	**R-5**	–	1	0.2
	**R-6**	–	1	0.3
	**R-7**	–	1.5	0.1
	**R-8**	–	1.5	0.2
	**R-9**	–	1.5	0.3
**S**	**S-1**	0.5	–	–
	**S-2**	1	–	–
	**S-3**	1.5	–	–
	**S-4**	2	–	–
	**S-5**	2.5	–	–
	**S-6**	3	–	–
	**S-7**	–	0.5	–
	**S-8**	–	1	–
	**S-9**	–	1.5	–
	**S-10**	–	2	–
	**S-11**	–	2.5	–
	**S-12**	–	3	–
	**S-13**	–	–	0.1
	**S-14**	–	–	0.2
	**S-15**	–	–	0.3

^a^V_ESF_,V_CSF_ and V_PF_ are the volume fraction of ESF, CSF and PF, respectively.

- Group O represents plain concrete, devoid of any fiber reinforcement.
- Group P focused on ESF and CSF, referred to as EC-HFRC; Group Q involved ESF and PF, named EP-HFRC; Group R combined CSF and PF, labeled CP-HFRC. Specifically, the volume fraction of ESF and CSF in these combinations varies between 0.5% and 1.5%, while that of PF ranges from 0.1% to 0.3%.
- Group S, on the other hand, represents the individual incorporation of all three fiber types. In this group, the volume fraction of ESF and CSF can vary within a broader range of 0.5% to 3.0%, while PF’s volume fraction remains within 0.1% to 0.3%.

### 2.2 Specimens

In each group, the type and volume fractions of reinforcing fibers were varied. Subsequently, based on the mix design specifications outlined in the previous section, distinct groups of concrete test specimens were fabricated for each group. Within each group, three identical parallel specimens were prepared to ensure consistency and repeatability. All the specimens were molded into non-standard cubes with a side length of 100mm.

For Group O, three plain concrete test specimens were made, with no fibers included. In Group P, Q and R, a total of 27 groups of specimens were prepared by incorporating varying combinations of two fiber types to create HFRC specimens. Each group comprised 9 groups of specimens, with three identical specimens per group. For Group S, a total of 15 groups of specimens were prepared, each incorporating a single type of fiber with varying volume fractions. This allowed for the examination of the effects of different fiber types and their varying concentrations on the properties of the concrete.

**Fig 3:**
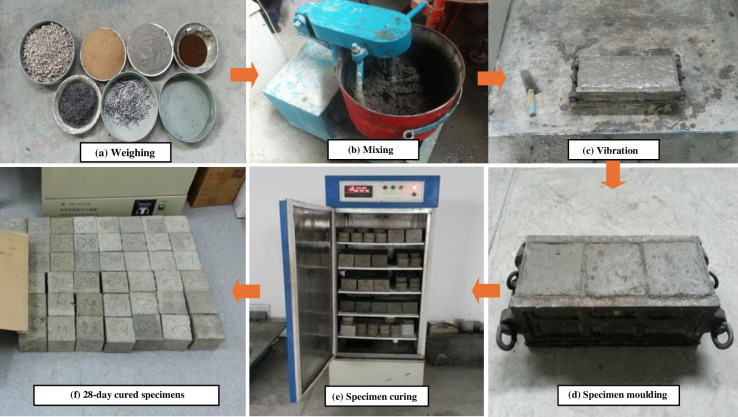
Test specimens making process. The specimens were thoroughly mixed and vibrated, subsequently demolded, and then transferred to standard curing conditions for an additional 27 days.

In this study, the preparation and the maintenance of the specimens are strictly in accordance with GB/T50107-2010 [30] and the previous research [31]. The specimens were cured at room temperature for 24 hours, then demolded and transferred to standard conditions (20 ± 2°C, 95% relative humidity) for curing for an additional 27 days. This curing process ensures that the concrete reaches its optimal strength and durability properties (Fig 3).

### 2.3 Test process

**Fig 4:**
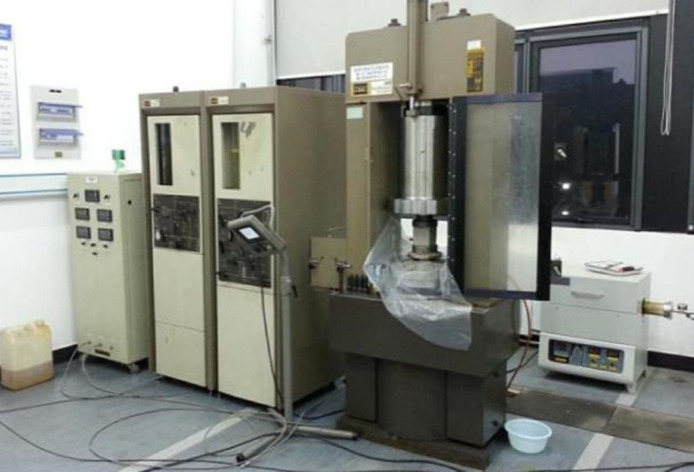
MTS816 hydrostatic test system.

All specimens in this study were subjected to unconfined compressive strength tests. The uniaxial compression tests were conducted using an electro-hydraulic servo testing machine (MTS816) with a maximum axial pressure of 1459 kN (Fig 4). The tests were performed strictly in accordance with Chinese standard GB/T50081-2019 [32]. To avoid eccentric loading and eliminate system relaxation, each specimen was preloaded to 10 kN three times before the formal test. During the formal test, since the cube compressive strength was not less than 60 MPa, a quasi-static loading method with a loading rate of 0.8 MPa/s was adopted until the specimen failed. The ultimate stress and photos of the failure state during the loading process were recorded. The average of the test results of three parallel specimens was taken as the 28-day compressive strength of each group.

## 3 Results and analysis

### 3.1 Results of compressive strength tests

The addition of a single type of fiber or a blend of two or more fiber types in concrete has the potential to enhance its performance. The experimental outcomes of the 28-day uniaxial compressive strength tests for all groups are presented in Table 4.

**Table 4:** Results of uniaxial compressive tests.

Groups	Group no.	*σ*_c_ (MPa)	*β*	*α*
**O**	**O**	60.63	1	1
**P**	**P-1**	63.75	1.051	1.043
	**P-2**	65.88	1.087	1.037
	**P-3**	66.44	1.096	1.014
	**P-4**	65.96	1.088	0.997
	**P-5**	67.53	1.114	0.982
	**P-6**	65.74	1.084	0.928
	**P-7**	67.34	1.111	1.000
	**P-8**	66.21	1.092	0.946
	**P-9**	64.97	1.072	0.900
**Q**	**Q-1**	61.58	1.016	1.016
	**Q-2**	66.51	1.097	1.090
	**Q-3**	64.84	1.069	1.084
	**Q-4**	66.85	1.103	1.020
	**Q-5**	70.81	1.168	1.072
	**Q-6**	65.7	1.084	1.015
	**Q-7**	66.66	1.099	0.999
	**Q-8**	72.34	1.193	1.076
	**Q-9**	65.83	1.086	0.999
**R**	**R-1**	61.57	1.016	1.019
	**R-2**	65.21	1.076	1.071
	**R-3**	61.04	1.007	1.023
	**R-4**	64.87	1.070	1.032
	**R-5**	70.07	1.156	1.107
	**R-6**	63.84	1.053	1.029
	**R-7**	65.76	1.085	1.015
	**R-8**	70.52	1.163	1.080
	**R-9**	63.79	1.052	0.998
**S**	**S-1**	60.95	1.005	1
	**S-2**	65.94	1.088	1
	**S-3**	67.15	1.108	1
	**S-4**	68.85	1.136	1
	**S-5**	67.05	1.106	1
	**S-6**	65.30	1.077	1
	**S-7**	60.81	1.003	1
	**S-8**	63.21	1.043	1
	**S-9**	65.17	1.075	1
	**S-10**	66.38	1.095	1
	**S-11**	65.47	1.080	1
	**S-12**	63.08	1.040	1
	**S-13**	60.27	0.994	1
	**S-14**	60.72	1.001	1
	**S-15**	59.49	0.981	1

To accurately assess the reinforcing and synergistic contributions of fibers in concrete, two dimensionless parameters were introduced: the fiber reinforcement coefficient *β* and the hybrid effect coefficient *α*. These definitions are in line with similar concepts previously utilized by researchers [33–34].

The fiber reinforcement coefficient *β* is defined as the percentage increase in compressive strength of concrete with fibers compared to the plain concrete compressive strength value, as expressed by Eq. (1).


$$\beta =\frac{f}{f_{m}}$$ (1)

where *β* is the fiber reinforcement coefficient of FRC; *f* is the compressive strength of FRC (MPa); *f*_*m*_ is the compressive strength of PC (MPa). For PC, *β* =  1.

The hybrid effect coefficient *α* is a quantitative index used to measure synergistic effect produced by the mixed fiber in concrete, that is, the improvement degree of the performance relative to two or more SFRC. For two types of fiber (A and B) mixed into concrete, the hybrid effect coefficient is represented by equation (2).


$$\alpha =\frac{\beta_{A,B}}{\beta_{A}\times \beta_{B}}$$ (2)

Where *α* is the hybrid effect coefficient; *β*_*A,B*_ is the fiber reinforcement coefficient of HFRC with fiber A and fiber B mixed; *β*_*A*_ is the fiber reinforcement coefficient of single mixed fiber A concrete (with the same mixing content as HFRC); *β*_*B*_ is the fiber reinforcement coefficient of single mixed fiber B concrete (with the same mixing content as HFRC). If *α*>1, it is a positive synergy effect, indicating that the performance of the concrete is enhanced, and the larger the value, the more significant; If α<1, it is a negative synergy effect, indicating that the performance of the concrete fails to meet the expectations, and may even shows a decrease in strength performance. For PC and SFRC, *α*=1. Table 4 presents the values of *β*, and *α* for all experimental groups.

### 3.2 Analysis of the reinforcement effect of mixed fibers on concrete

#### 3.2.1 Influence of fiber mixing on the reinforcement effect of concrete.

The effect of fiber mixing on the reinforcing effect of concrete can be clearly observed by analyzing the detailed test data presented in Table 4. The benchmark 28-day compressive strength of PC is 60.63 MPa. Taking the test of group Q-3 as an example, when the rate of ESF mixing is 0.5% and the rate of PF mixing is 0.3%, the 28-day compressive strength of the EP-HFRC is enhanced to 64.84 MPa. Compared with the benchmark PC, the *β of* group Q-3 is as high as 1.069, which indicates that the fiber admixture significantly enhanced the concrete’s compressive properties.

In contrast, the compressive strength of the group S-1 with 0.5% ESF alone was 60.95 MPa, and the value of *β* was only 1.005, which showed a limited enhancement. The compressive strength of the group S-15 mixed with 0.3% PF was 59.49 MPa with the *β* of 0.981. This indicates that the single mix of polypropylene fibers did not have a significant effect on the improvement of the compressive strength of concrete.

**Fig 5:**
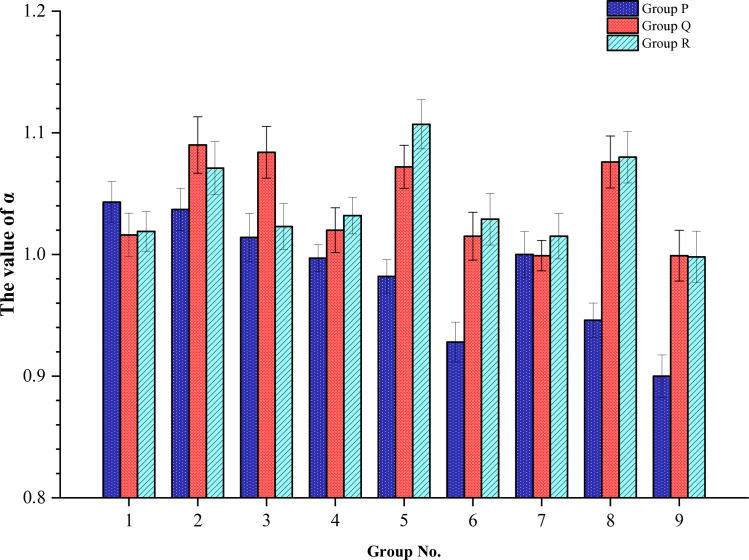
The value of *α* for Group P, Q, and R.

From the perspective of the *α*, it is observed that some coefficients are greater than 1 while others are less than 1, indicating both positive and negative hybrid effects, as shown in Fig 5. However, the *α* values for the majority of HFRC exceed 1. In particular, the *α* of group R-5 was the largest, reaching 1.107. This suggests that hybrid fibers can achieve a positive confounding effect compared to SFRC, thereby improving the static compressive strength of concrete to some extent.

#### 3.2.2 Influence of fiber type on the reinforcement effect of concrete.

In investigating the impact of fiber incorporation on the compressive properties of concrete, it was observed that the degree of enhancement varied considerably depending on the fiber type. For Group P tests, the hybrid effect coefficient was greater than 1 when the ESF mixing rate was 0.5%, showing a positive hybrid effect. However, as the ESF mixing rate was increased to 1% and 1.5%, the hybrid effect coefficient decreased to less than 1, indicating a negative hybrid effect. A comparable pattern was observed in the Group Q and R tests. As illustrated in Fig 5, the hybrid effect coefficient exhibited a gradual decline as the fiber mixing rate increased. This may be attributed to the propensity of the fibers with a high content to agglomerate during the mixing process.

Therefore, in order to obtain a positive hybrid effect, the ESF ratio of EC-HFRC should be controlled at about 0.5%, and the total fiber content should not exceed 2%. In contrast, EP-HFRC exhibits superior hybrid effect due to the distinctive properties of its constituent fibers - high elastic modulus of ESF and low elastic modulus and high ductility of PF. The data obtained from the experimental trials indicate that the hybrid effect coefficients of the majority of the EP-HFRC test groups are above 1, with only two exceptions, Q-7 and Q-9, which are slightly below 1. These findings confirm that significant reinforcement effects can be achieved through fiber hybridization. Similarly, CP-HFRC displayed excellent hybridization performance, with hybrid effect coefficients greater than 1 for all test groups, with the exception of the R-9 group. Further comparison of the mean hybrid effect coefficients of the test groups revealed that the hybridization effects of CP-HFRC (1.042) and EP-HFRC (1.041) were significantly better than those of EC-HFRC (0.983). In other words, the combination of steel and polypropylene fibers results in a more effective reinforcement. The rationale behind this phenomenon can be attributed to the micro-sized polypropylene fibers’ ability to bridge the micro-cracks in concrete. This is due to the fibers’ good dispersibility and bonding properties with the matrix. The synergistic interactions between different types of fibers exert a positive hybrid effect in increasing the overall strength of the concrete [35].

Based on the above analysis, EP-HFRC is the optimal fiber mixing type selection when the steel fiber mixing rate is 0.5%, while CP-HFRC becomes a more optimal fiber mixing type selection when the steel fiber mixing rate is increased to 1% and 1.5%.

#### 3.2.3 Optimal fiber mix for maximizing compressive strength.

**Fig 6:**
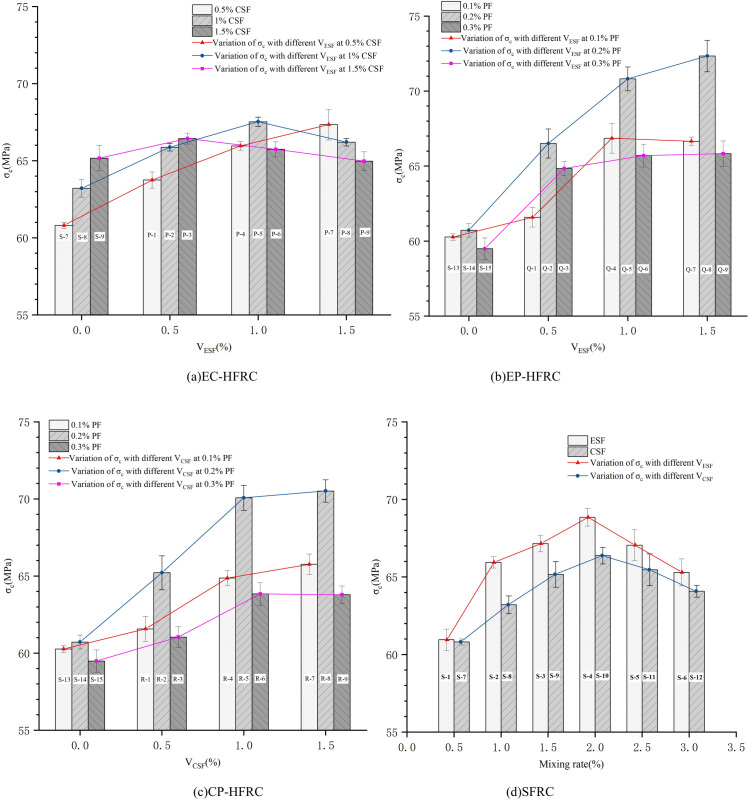
Variation in compressive strength of EC-HFRC, EP-HFRC, CP-HFRC and SFRC with changes in fiber content. (a) The red, blue, and magenta line graphs illustrate the impact of varying the volume of ESF on the static compressive strength of HFRC, with the CSF volume set at 0.5%, 1% and 1.5%, respectively. (b) The red, blue, and magenta line graphs show the static compressive strength of HFRC varied depending on the volume of ESF, with the PF volume set at 0.1%, 0.2% and 0.3%, respectively. (c) The red, blue, and magenta line graphs demonstrate that the static compressive strength of HFRC varies in accordance with the volume of CSF, with the PF volume set at 0.1%, 0.2%, and 0.3%, respectively. (d)The red and blue line graphs illustrate the variation in compressive strength of HFRC corresponding to different volumes of ESF and CSF, respectively.

In order to deeply investigate the optimum fiber mixing ratio for HFRC, full scale fiber doping tests were conducted. In this study, the HFRC properties of ESF and CSF at 0.5%, 1%, and 1.5% doping, respectively, and the HFRC properties of PF at 0.1%, 0.2%, and 0.3% doping were tested. Based on the test data, the line graphs of the effect of fiber volume ratio on the static compressive strength of HFRC were plotted, as shown in Fig 6.

Through meticulous analysis of the line graphs, it is observed that the compressive strength of HFRC increases significantly with the increase in the content of the other fiber at 0.5%CSF (Fig 6(a)). However, when the ratio of CSF reaches 1% and 1.5%, respectively, the reinforcement effect of the compressive strength gradually diminishes and even shows a slightly decreasing trend.

As demonstrated in Fig 6(b), it is evident that the compressive strength of EP-HFRC attains its maximum value when the PF content is set at 0.2%, in comparison to the values observed at 0.1% and 0.3%. The strength increases in proportion to the ESF content. When the ESF content is 1.5%, the compressive strength reaches its maximum value of 72.34 MPa. Consequently, the optimal fiber ratio of EP-HFRC is determined to be 0.2% PF and 1.5% ESF.

As demonstrated in Fig 6(c), it can be concluded that when the PF content is 0.2%, the compressive strength of CP-HFRC reaches its maximum value in comparison with the PF dosages of 0.1% and 0.3%. Furthermore, it is evident that the compressive strength increases with an increase in the CSF dosage. It is notable that when the CSF content is 1.5%, the compressive strength reaches its maximum value of 70.52 MPa. Consequently, the optimal fiber ratios for CP-HFRC are determined to be 0.2% PF and 1.5% CSF.

The analysis of Fig 6(d) reveals that the incorporation of single-doped steel fibers into concrete can enhance its compressive strength, albeit to a limited extent. The enhancement in compressive strength is observed to peak at 13.6% when the ESF content is set at the 2% level.

**Fig 7:**
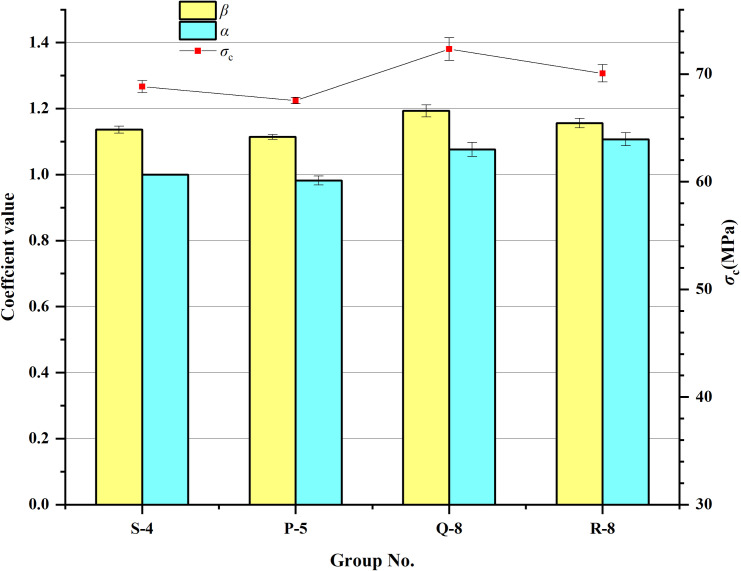
Comparison of the performance parameters for the peak compressive strength corresponding to the test groups. *β* is the fiber reinforcement coefficient of fiber mixing concrete. *α* is the hybrid effect coefficient. The highest peak compressive strength was observed in the group Q-8, which exhibited the greatest fiber mixing effect coefficient.

It is notable that four groups of tests, designated S-4, P-5, Q-8, and R-8, demonstrate the peak compressive strength of HFRC, as illustrated in Fig 7. Among them, S-4 composition with 2.0% ESF alone is the best choice when steel fibers are doped alone. Group P-5 demonstrates the advantage of mixing ESF and CSF, with the compressive strength of HFRC reaching its maximum value when both ESF and CSF are 1%. This mixing combination is therefore considered optimal in terms of engineering strength demand. However, the value of *α* is slightly lower than 1, indicating that the efficiency of strength enhancement is not optimal.

Similarly, for EP-HFRC and CP-HFRC, the Q-8 and R-8 groups reached peak compressive strengths with optimum fiber mixes of 0.2% PF and 1.5% ESF, and 0.2% PF and 1.5% CSF, respectively. Meanwhile, the fiber mixing effect coefficients are both greater than 1.16, indicating that these two groups of mixed fiber combinations exert the maximum efficiency of fibers, and the concrete reinforcement effect is maximized, thus achieving the best economic efficiency.

Therefore, theoretically, the optimal fiber combination identified for enhancing compressive strength consists of 0.2% PF in conjunction with 1.5% ESF within EP-HFRC.

### 3.3 Failure process and modes

**Fig 8:**
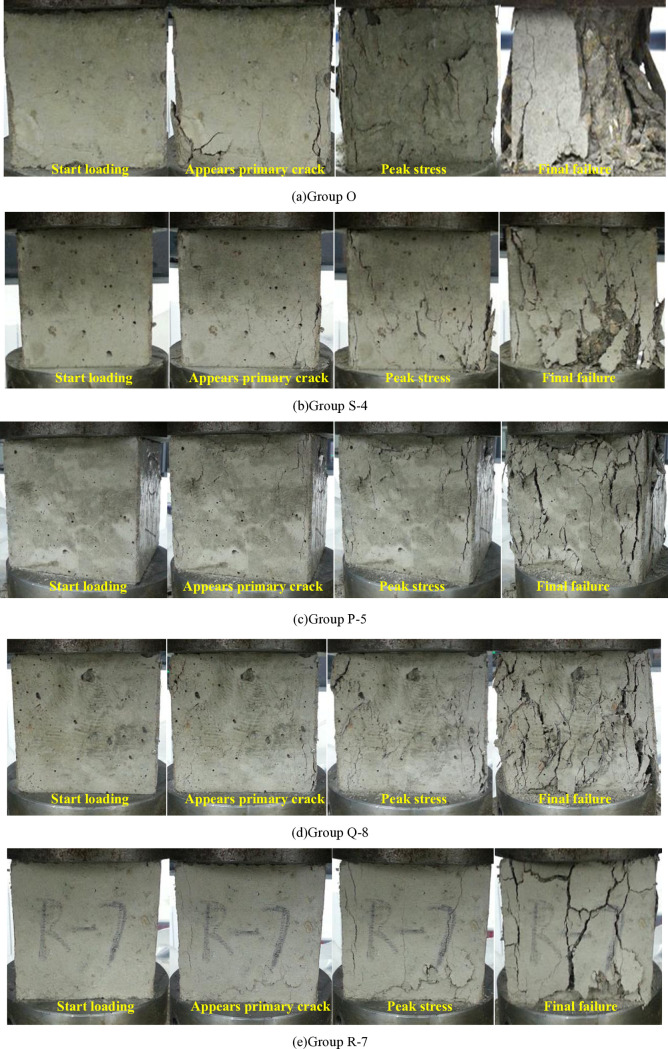
Failure process and modes. Four stages of the five typical specimens corresponding to each group from the beginning of loading to the initial crack generation, to the peak stress, and finally to the final failure were listed.

The MTS816 hydrostatic test system was employed to observe the damage evolution of different types of concrete, including PC, single-mixed steel fiber concrete, EC-HFRC, EP-HFRC, and CP-HFRC, during the compression process. Fig 8 lists the four stages of the five typical specimens corresponding to each group, from the beginning of loading to the initial crack generation, to the peak stress, and finally to the final failure. The failure modes of all specimens are classified as satisfactory failures in accordance with BS EN 12390-3:2002 [36].

It can be seen from Fig 8(a) that for PC, many fine cracks appear gradually after the loading starts. When the peak stress is reached, these cracks gradually expand and intersect with each other, resulting in cracks that run through the surface of the specimen, and causing the peeling of the concrete surface, and the transverse volume gradually expands. Continue loading, large pieces of concrete fall off, and the interior of the concrete is exposed. Upon reaching the final stage of failure, almost half of the concrete collapses. This is a typical failure process of brittle materials. This result is consistent with previous research results [37].

As can be seen from Fig 8(b), for steel fiber reinforced concrete with single admixture, the presence of steel fibers restricts the rapid expansion of cracks. From the failure process, it can be seen that compared with the failure of PC, after the steel fiber reinforced concrete with single admixture reaches the peak load, the through cracks are reduced, and there is no large-scale falling off during the final failure. The steel fibers are pulled out of the cracks or broken, which to a certain extent improves the brittle failure characteristic of the concrete. This failure characteristic is consistent with previous research results [38].

As can be seen from Fig 8(c)–(e), for the three types of HFRC, namely EC-HFRC, EP-HFRC, and CP-HFRC, cracks are fine and relatively few in number. Once cracks appear, the hybrid fibers begin to take effect. The combination of fibers with different elastic modulus and geometric shapes exerts a synergistic effect within the concrete, generating a large bonding force between the hybrid fibers and the concrete matrix, which increases the energy consumed during fiber extraction. There is no fragmentation of fragments during the final failure, but rather a large number of cracks and peeling phenomena, and the specimens still maintain a good shape. This damage pattern is consistent with previous research results [22].

Therefore, compared with single steel fiber admixture and no fiber admixture, the mixing of hybrid fibers can significantly impact the failure mode of concrete by mitigating the brittle failure characteristic of concrete and imparting a degree of plastic failure characteristic. HFRC specimens can still maintain a relatively intact shape when finally destroyed. The order of damage speed of the specimens from fast to slow is: PC>  SFRC>  EC-HFRC>  CP-HFRC>  EP-HFRC.

## 4 Strength estimation of fiber reinforced concrete

### 4.1 Strength estimation of single-fiber reinforced concrete

Previous analyses have revealed that steel fibers exhibit a particularly pronounced effect on enhancing the strength of concrete. Consequently, this study focuses on predicting the uniaxial compressive strength of concretes with singly mixed steel fibers (ESF and CSF), as well as HFRC. According to the mechanics theory of composites, composites are composed of multiple phases, and their elastic modulus, strength, and other indicators can be calculated based on the superposition principle of individual phases [39]. FRC is a type of composite material, and the application of this theory is premised on the following assumptions: (1) both fibers and the matrix are considered as elastic deformation bodies, and their deformations are equal in the transverse direction; (2) there are minute strains between fibers and the matrix; (3) fibers are aligned in the same direction as the stress, and this alignment is uniform and continuous; (4) there is no relative sliding between fibers and the matrix [39].

Under the satisfaction of the basic assumptions, the fibers within the concrete, when subjected to stress along the fiber direction, can have their average stress calculated using equation (3) [39].


$$\sigma =\sigma_{m}\left(1-{\text{V}}_{f}\right)+\sigma_{f}V_{f}$$ (3)

Where, *σ* is the stress of FRC; *σ*_*m*_ is the stress of the concrete matrix; *σ*_*f*_ is the stress within the fibers, typically the absolute value of the average tensile stress in the fibers; and *V*_*f*_ is the fiber volume fraction.

**Fig 9:**
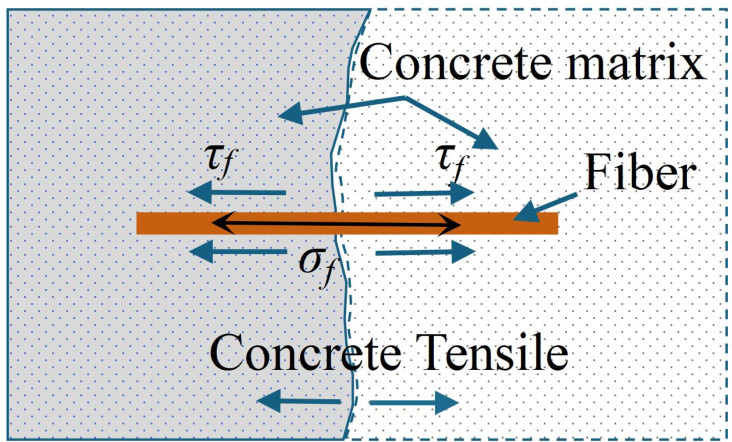
The schematic diagram of the pullout of a single fiber during the compression of the specimen. *τ*_*f*_ is the shear stress at the interface between the fiber and the concrete matrix.


Fig 9 illustrates the schematic diagram of the pullout of a single fiber. By balancing the forces and assuming that the embedded length of the fiber is half of its total length, a derivation can be made as follows:

$$\sigma_{f}=2\frac{l_{f}}{d_{f}}\tau_{f}$$ (4)

where, *l*_*f*_ is the fiber length; *d*_*f*_ is the fiber diameter; and *τ*_*f*_ is the shear stress at the interface between the fiber and the concrete matrix.

Substitute equation (4) into equation (3) to obtain:


$$\sigma =\sigma_{m}\left(1-{\text{V}}_{f}\right)+2\frac{l_{f}}{d_{f}}\tau_{f}V_{f}$$ (5)

**Fig 10:**
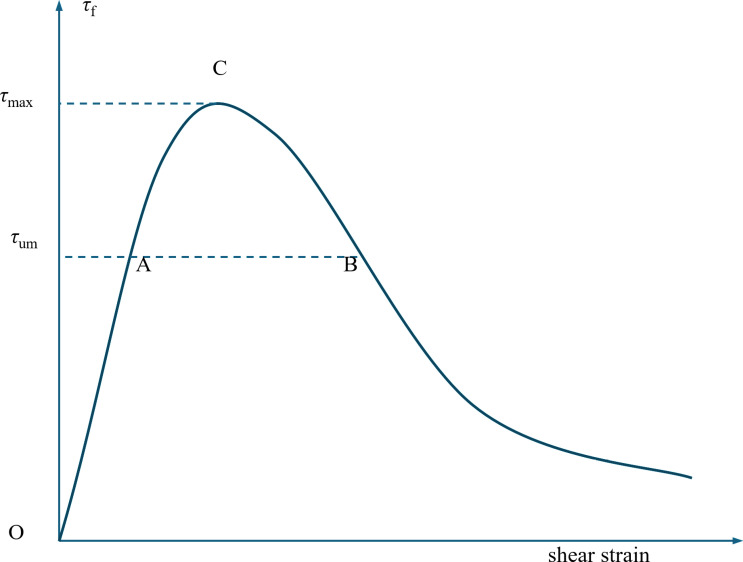
Variation of shear stress during the pullout of a single fiber with respect to the shear strain [41]. *τ*_um_ represents the mean shear stress at the interface between all fibers and the matrix at fiber reinforced concrete (FRC) stress limit state. Similarly, *τ*_max_ denotes the maximum shear stress in the same cases. The shear stress increases with the increasing deformation of the concrete until it reaches *τ*_max_, and then decreases as deformation continues to increase.

Liu et al. [40] and Wang et al. [41] conducted pullout tests on individual fibers embedded in concrete matrices and found that as the pullout displacement increased, the pullout force also increased. After reaching a maximum value, the pullout force decreased. When converted to the shear stress at the interface between the fiber and the concrete, the same pattern was observed, as shown in Fig 10 [41].

As illustrated in Fig 10, the shear stress at the interface between the fiber and the concrete matrix undergoes a change during the deformation process of the FRC. Initially, the shear stress increases with the increasing deformation of the concrete until it reaches a maximum shear stress, designated as *τ*_*max*_. Subsequently, as deformation continues to increase, the shear stress decreases.

In the actual mixing process of FRC, fibers are randomly distributed. During failure, phenomena such as fibers being pulled out of the concrete or breaking can occur, which do not conform to basic assumptions. Therefore, the effective coefficient of fiber action *η* was introduced, and the strength of the FRC at the limit state of stress can be expressed as follows:


$$\sigma_{c}=\sigma_{m}\left(1-{\text{V}}_{f}\right)+\eta \frac{l_{f}}{d_{f}}\tau_{um}V_{f}$$ (6)

Where, *σ*_*c*_ is the ultimate uniaxial compressive strength of the FRC; *η* is the effective coefficient of fiber action; and *τ*_*um*_ is the average shear stress at the interface between all fibers and the matrix at FRC stress limit state.

The average shear stress *τ*_*um*_ at the fiber-matrix interface under the ultimate stress state of FRC varies with the increase of the fiber volume fraction *V*_*f*_. The law of this variation is as follows:

(1) Given the random orientation of fibers within the matrix, it is unlikely that all fibers will simultaneously attain the ultimate shear state point C (refer to Fig 10) when FRC reaches its ultimate stress state. When *V*_f_ is low, the limited number of fibers in the matrix exerts weaker constraints on matrix deformation. Consequently, as the FRC approaches its ultimate stress state, the fibers undergo significant shear deformation, with most reaching state point B. This results in a relatively low average shear stress at the fiber-matrix interface, denoted as *τ*_*um*_. Conversely, when *V*_*f*_ is high, the increased number of fibers in the matrix imposes stronger constraints on matrix deformation. As the concrete reaches its ultimate stress state, the fibers experience less shear deformation, with most reaching state point A. However, this does not significantly elevate *τ*_*um*_. The optimal group arises when *V*_*f*_ is judiciously balanced, enabling the ultimate compressive stress state of the concrete to coincide precisely with the ultimate shear state of the fibers. Under these conditions, most fibers attain state point C, representing the most desirable and efficient state, where *τ*_*um*_ is optimized.
(2) Due to the agglomeration effect of fibers, when *V*_*f*_ becomes excessively high, the density of fibers in the concrete increases substantially, reducing the inter-fiber spacing. This leads to overlapping stress influence zones and mutual interactions among fibers, ultimately diminishing the individual pullout resistance of each fiber. Furthermore, the dense fiber distribution promotes interference, crossovers, and overlapping among fibers, rendering the reinforcement effect non-linear and not simply additive. In some cases, this can even yield negative consequences. Consequently, an overly large *V*_*f*_ does not necessarily prioritize the enhancement of concrete properties, and under such conditions, *τ*_*um*_ remains relatively low. Thus, the optimal *V*_*f*_ must be carefully determined to balance fiber reinforcement benefits against potential drawbacks of excessive fiber density.

It is evident that as *V*_*f*_ increases from a low to a high value, *τ*_*um*_ first undergoes an increase and then a decrease. This characteristic trend can be aptly described by a parabolic equation (7), which captures the initial enhancement in *τ*_*um*_ due to the beneficial effects of fiber reinforcement, followed by a decline as excessive fiber density leads to negative interactions and reduced efficiency [21].


$$\tau_{um}=a+b{\text{V}}_{f}-cV_{f}^{2}$$ (7)

Where, a, b, and c are all pending constants greater than 0.

By substituting the formula (7) into the formula (6), the following result is obtained:


$$\sigma_{c}=\sigma_{m}+\left(\eta a-\frac{d_{f}}{l_{f}}\sigma_{m}\right)\left(\frac{l_{f}}{d_{f}}V_{f}\right)+\eta \frac{d_{f}}{l_{f}}b{\left(\frac{l_{f}}{d_{f}}V_{f}\right)}^{2}-c\eta \frac{d_{f}^{2}}{l_{f}^{2}}{\left(\frac{l_{f}}{d_{f}}V_{f}\right)}^{3}$$ (8)

In order to analyze the effect of fiber geometry and volume fraction on the reinforcement, the reinforcement index (*R*_*I*_) of the fiber was introduced, defined as [21,42]:


$$R_{I}=\frac{l_{f}}{d_{f}}V_{f}$$ (9)

The calculated results of *R*_*I*_ of single doped fibers in Group S are presented in Table 5. The fiber length and diameter calculated in *R*_*I*_ were taken as their respective median values.

**Table 5:** Calculation of *R*_*I*_ and *R*_*IH*_ for Group P, Q, R, and S.

Index	Group	Group No.											
		1	2	3	4	5	6	7	8	9	10	11	12
*R*_*I*_	**S**	0.23	0.47	0.70	0.93	1.17	1.40	0.34	0.68	1.02	1.36	1.70	2.04
*R*_*IH*_	**P**	0.57	0.91	1.25	0.81	1.15	1.49	1.04	1.38	1.72	–	–	–
	**Q**	0.24	0.24	0.24	0.47	0.47	0.48	0.7	0.71	0.71	–	–	–
	**R**	0.34	0.35	0.35	0.68	0.69	0.69	1.02	1.03	1.03	–	–	–

The introduction of *R*_*I*_ allowed for the abbreviation of equation (8), as follows:


$$\sigma_{c}=\sigma_{m}+AR_{I}+BR_{I}^{2}-CR_{I}^{3}$$ (10)

Where, A, B, and C are undetermined constants that correspond to the respective terms in equation (10). A, B, and C are calculated as:


$$A=\eta a-\frac{d_{f}}{l_{f}}\sigma_{m}$$ (11)

$$B=\eta \frac{d_{f}}{l_{f}}b$$ (12)

$$C=c\eta \frac{d_{f}^{2}}{l_{f}^{2}}$$ (13)

**Fig 11:**
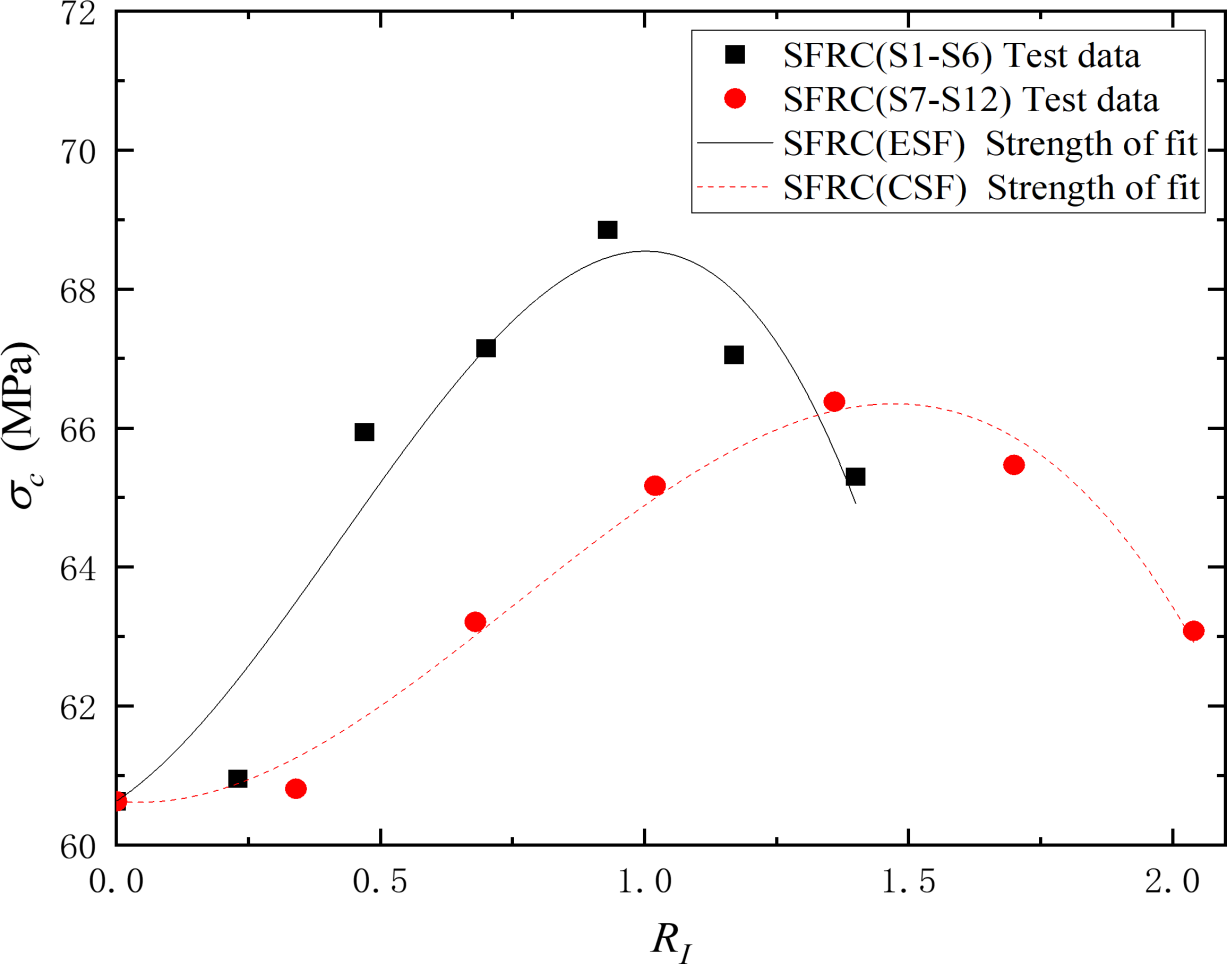
Comparison of experimental and fitted strengths of steel fiber reinforced concrete. *σ*_c_ is the ultimate uniaxial compressive strength of the FRC. *R*_*I*_ is the fiber reinforcement index. The solid black squares represent the experimental strength values of group S1-S6. The solid red circles represent the experimental strength values of group S7-S12. The solid black line shows fitted strength of end-hooked steel fiber reinforced concrete. The red dotted line shows fitted strength of corrugated steel fiber reinforced concrete.

The experimental data from groups S-1 to S-12 were fitted using equation (10), and the results are presented in Table 6 and Fig 11. It can be observed that the fitted strength values, which are calculated using *R*_*I*_, exhibits a consistent trend with the experimental strength values, with relatively small errors. *R*_*I*_ has been proved to be a suitable parameter of fibers for describing the mechanical properties of FRC.

**Table 6:** Fitting parameters for strength estimation of steel fiber reinforced concrete.

Groups	A	B	C	*R* ^*2a*^
**S1**-**S6**	5.0706	13.5451	10.7005	0.9285
**S7**-**S12**	-0.7405	8.9217	3.9260	0.9847

^a^*R*^*2*^ is the coefficient of determination, reflects the goodness of fit of the model.

### 4.2 Hybrid fiber reinforced concrete strength estimation

When there are two or more types of fibers in HFRC, calculating the fiber reinforcement index using equation (9) might present difficulties. It is proposed that a weighted method be adopted to define the fiber reinforcement index. That is,


$$R_{IH}=\sum_{i}^{n}R_{Ii}$$ (14)

Where, *i* is the sequence number of the fiber type; *R*_*Ii*_ is the reinforcement index of the *i*^*th*^ type of fiber.


$$R_{I\text{i}}=V_{fi}\frac{l_{fi}}{d_{fi}}\frac{E_{i}}{E_{SF}}$$ (15)

Where, *V*_*fi*_ is the volume fraction of the *i*^*th*^ type of fiber; *l*_*fi*_ is the length of the *i*^*th*^ type of fiber; *d*_*fi*_ is the diameter of the *i*^*th*^ type of fiber; *E*_*i*_ is the elastic modulus of the *i*^*th*^ type of fiber; *E*_*sf*_ is the elastic modulus of the steel fiber. Similar definitions have been proposed in previous research [21,22], and our definition eliminates the shape factor coefficient since the influence of fiber shape is already considered in the undetermined constant. The calculation results of *R*_*IH*_ of the hybrid fibers in groups P, Q, and R are shown in Table 5.

Substitute *R*_*IH*_ for *R*_*I*_ in equation (10). In order to consider the effect of fiber interaction, the coupling coefficient of the hybrid fibers, *k*, is introduced to obtain:


$$\sigma_{c}=\sigma_{m}+AkR_{IH}+B{\left(kR_{IH}\right)}^{2}-C{\left(kR_{IH}\right)}^{3}$$ (16)

or


$$\sigma_{c}=\sigma_{m}+A^{\prime }R_{IH}+B^{\prime }R_{IH}^{2}-C^{\prime }R_{IH}^{3}$$ (17)

Where, A’, B’, and C’ are undetermined constants, which are calculated as:


$$A^{\prime }=kA$$ (18)

$$B^{\prime }=k^{2}B$$ (19)

$$C^{\prime }=k^{3}C$$ (20)

**Fig 12:**
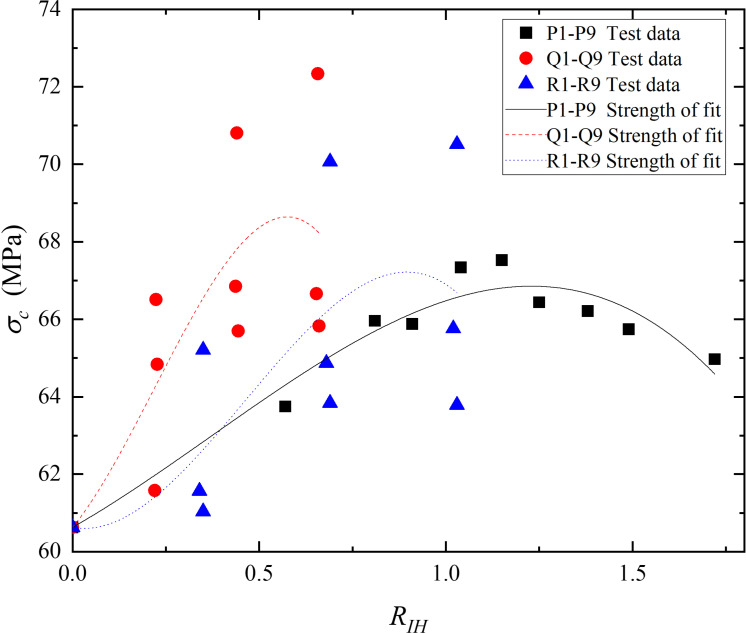
Comparison of experimental and fitted strengths for HFRC. *R*_*IH*_ is the hybrid fiber reinforcement index. The solid black squares represent the experimental strength values of group P1-P9. The solid red circles represent the experimental strength values of group Q1-Q9. The solid blue triangles represent the experimental strength values of group R1-R9. The solid black line shows fitted strength of P1-P9. The red dotted line shows fitted strength of Q1-Q9. The blue dotted line shows fitted strength of R1-R9.

Then, apply equation (17) to fit the experimental data from groups P, Q, and R, the results are shown in Table 7 and Fig 12. It can be observed that when the differences among hybrid fiber types are not significant, there is a good correlation between the experimental strength and the estimated values, with correlation coefficients close to 1, as in EC-HFRC. Conversely, when the differences among hybrid fiber types are substantial, the correlation between the experimental strength and the estimated values is poorer, evidenced by lower correlation coefficients, as in EP-HFRC and CP-HFRC.

**Table 7:** Fitting parameters for the strength estimation of HFRC.

Group	A’	B’	C’	*R* ^*2*^
**P1-P9**	5.4861	3.4369	3.0751	0.93599
**Q1-Q9**	11.7264	31.9578	48.8943	0.54775
**R1-R9**	-1.7385	28.5507	20.5404	0.46636

In summary, the strength estimation methodology proposed in this study proves to be viable for steel SFRC and EC-HFRC with high accuracy. *R*_*I*_ has been proved to be a suitable parameter of fibers for describing the mechanical properties of FRC. This method offers a reliable tool for engineers and researchers seeking to predict the mechanical properties of these advanced concrete materials, thereby facilitating their optimal utilization in various construction applications.

## 5 Conclusions and prospects

In this research, comprehensive compressive strength testing was conducted on PC, SFRC and HFRC. The findings demonstrate that the incorporation of fibers significantly influences the compressive strength of concrete. Not only does the type of fiber play a crucial role, but also the proportion of fiber content. The integration of hybrid fibers was observed to enhance not just the strength but also the concrete’s resistance to cracking. Utilizing the theory of composite materials mechanics, a predictive model for the compressive strength of FRC has been derived. This study provides valuable insights into optimizing fiber combinations and dosages to achieve improved mechanical performance in concrete applications. The following conclusions were obtained from this study:

- The mixing of fibers significantly increased the compressive strength of concrete under the test conditions. The fiber reinforcement coefficients of HFRC were all greater than 1, indicating different degrees of compressive strength enhancement compared to PC and SFRC. Ranked by the mean value of the hybrid effect coefficient, CP-HFRC (1.042) was slightly higher than EP-HFRC (1.041), and both were significantly better than EC-HFRC (0.983). In addition, the hybrid effect coefficient of EC-HFRC gradually decreases with the increase of fiber doping, while the hybrid effect of EP-HFRC and CP-HFRC is more significant.
- Fiber type is the key factor affecting the static compressive performance of HFRC. It was found that the integration of steel and polypropylene fibers resulted in a more effective reinforcement for the compressive strength of HFRC compared to the use of steel fibers alone. The EP-HFRC exhibited optimal performance when the steel fiber ratio was 0.5%, while the CP-HFRC demonstrated better performance at 1% and 1.5% steel fiber ratios.
- Fiber content has an important effect on the compressive properties of HFRC. For EC-HFRC, the combination of 1% ESF and 1% CSF is recommended from the engineering strength point of view. For EP-HFRC and CP-HFRC, the optimal fiber mixing combinations were 0.2% PF with 1.5% ESF and 0.2% PF with 1.5% CSF, respectively.
- By comparing and analyzing the damage process and morphology of specimens of HFRC, SFRC and PC, it is found that the mixing of hybrid fibers can significantly impact the failure mode of concrete by mitigating the brittle failure characteristic of concrete and imparting a degree of plastic failure characteristic.
- A mathematical model has been developed on the basis of the theory of composite materials mechanics to predict the uniaxial compressive strength of single and hybrid steel FRC. This model has been compared and analyzed with experimental data, demonstrating a good degree of fit. This achievement provides theoretical support for the design and optimization of FRC.

In this study, the fabrication and preparation of fibers and specimens is subject to human error, which has the potential to affect the accuracy of the results. The predictive model proposed in this paper is based on the theory of composite materials. In order to simplify the theoretical derivation and calculation, the following assumptions are made: the fibers are uniformly distributed and continuous in the concrete, the arrangement direction of the fibers is the same as the direction of the stress, and the fibers are not pulled out or broken when the concrete cracks. This assumption is not in alignment with the actual state of fibers in concrete. Despite the introduction of the fiber action coefficient for the purpose of correction, the model remains subject to bias. The prediction model outlined in this paper demonstrates a strong correlation with the compressive strength values of steel FRC. However, further exploration is necessary to extend its application to concrete mixed with steel and polypropylene fibers. This study focuses on the compressive strength of HFRC under uniaxial stress conditions. Further exploration is necessary to investigate other properties of HFRC, including crack resistance, durability, and impact resistance, as well as its application under complex environmental conditions and multiple stress loading conditions. This will enhance the potential application of HFRC in practical engineering.

**Table:** Nomenclature

HFRC	Hybrid fiber reinforced concrete
FRC	Fiber-reinforced concrete
PC	Plain concrete
SFRC	Single-fiber reinforced concrete
ESF	End-hooked steel fibers
CSF	Corrugated steel fibers
PF	Polypropylene fibers
EC-HFRC	HFRC doped with ESF and CSF
EP-HFRC	HFRC doped with ESF and PF
CP-HFRC	HFRC doped with CSF and PF
V_ESF_	Volume fraction of ESF
V_CSF_	Volume fraction of CSF
V_PF_	Volume fraction of PF
*β*	Fiber reinforcement coefficient
*α*	Hybrid effect coefficient
*f*	Compressive strength of FRC
*f*_*m*_	Compressive strength of PC
*β*_*A,B*_	Fiber reinforcement coefficient of HFRC with fiber A and fiber B mixed
*β*_*A*_	Fiber reinforcement coefficient of single mixed fiber A concrete
*β*_*B*_	Fiber reinforcement coefficient of single mixed fiber B concrete
*σ*_c_	Compressive strength
*R*_*I*_	Fiber reinforcement index
*σ*	Stress of FRC
*σ*_m_	Stress of the concrete matrix
*σ*_*f*_	Stress within the fibers
*V *_*f *_.	Fiber volume fraction
*l*_*f*_	Fiber length.
*d*_*f*_	Fiber diameter
*τ*_*f*_	Shear stress at the interface between the fiber and the concrete matrix
*τ*_*max*_	Maximum shear stress
*σ*_*c*_	Ultimate uniaxial compressive strength of the FRC
*η*	Effective coefficient of fiber action
*τ*_*um*_	Average shear stress at the interface between all fibers and the matrix
*R*_*I*_	Fiber reinforcement index
*R* ^*2*^	Coefficient of determination
*R*_*IH*_	Weighted fiber reinforcement index
*E*_*i*_	Elastic modulus of the *i* ^*th*^ type of fiber.
*E*_*sf*_	Elastic modulus of the steel fiber
*k*	Coupling coefficient of the hybrid fibers
